# Unrecognized ambiguities in validity of intervention research: an example on explicit phonics and text-centered teaching

**DOI:** 10.3389/fpsyg.2014.01535

**Published:** 2015-01-07

**Authors:** G. Brian Thompson

**Affiliations:** School of Education, Victoria University of WellingtonWellington, New Zealand

**Keywords:** teaching reading, beginner reading, low reading attainment, instruction intervention, intervention research method, reading acquisition theory, explicit phonics, text-centered teaching

Tse and Nicholson (2014) have tested a small-group instructional intervention that they propose as a modification to enhance reading progress among low attainment 6-year-olds in a “text-centered” teaching approach. The authors (T&N) cite a Ministry of Education (2003) handbook to describe this approach. It has four main components (pp. 91–101): (i) Teacher Reading of texts to listening children, (ii) Shared Reading in which the children engage in watching the text print (“Big Books”) as the teacher shows how it matches the spoken text, (iii) Guided Reading in which there is detailed teacher support of the individual children's attempts at reading a text (e.g., for “using word-level information to decode new words” p. 97), (iv) Children's Independent Reading of texts (with minimal errors) by themselves for individual levels and interests. This report of T&N, however, lacked evidence about what the children received of each of these components prior to, and concurrent with, the intervention study. Without such evidence we cannot tell in what way the instructional interventions were the same, different from, or in conflict with other instruction received.

T&N's proposed modification to the Shared Reading component was to combine it with systematically taught explicit phonics (a “sounding out” procedure in which the child pronounces successive sounds of letters of a word to generate an oral reading response). For theoretical justification of this modification, T&N cited some of the claims of Gough and Hillinger (1980) but omitted others, that phonics “gives the child artificial rules …… to learn the real rules” (p. 192), which “are unconscious and implicit” (p. 187). This implies that phonics is a heuristic procedure for initial instruction but subsequently discarded without any disadvantage [although Thompson et al. (2009) found evidence to the contrary]. Neither T&N nor their citation of Gough and Hillinger provide justification for the particular phonics rules (e.g., final *e*-marker of “long” vowels) and corresponding sounds (e.g., for vowel digraphs) selected for instruction (T&N, Table 2) of these 6-year-olds with word reading test ages in the lower half of the normative distribution, and a mean aural vocabulary test age of 4 years 8 months (determined from BPVT norms using raw scores in T&N, Table 4). T&N found no effect of their intervention on the children's aural vocabulary but were silent on why the overall text-centered approach, with their modification, would be suitable for children with an apparent large developmental lag in understanding spoken English.

T&N gave no report of the opportunities that the items of the pre-and post-test measures provided for children to use the taught phonics procedures. Interpretation of results for each measure depends upon the extent to which these opportunities were provided; and for comparison between measures, whether such opportunities were equal or different. For each reading measure the writer determined the percentage of word items that provided this opportunity among items in the applicable reading-level range. For example, this was 34% of items providing opportunities in the decoding skills measure. It was, however, 16% in the isolated word reading, and in this there were also 16% that provided conflicting opportunities because the taught procedures could not work (e.g., final*-e* marker of “long vowels” in the words *one*, *love*). The decoding skills items had no conflicting opportunities. Hence, any superior score gains for this measure could be just an artifact of more (workable) opportunities. Another unbalanced feature of the design is noted. The phonics procedures demonstrated to the children were followed up by their individual attempts at weekly “quizzes” (T&N, Table 3; Figure 4). There were no similar individual opportunities involving text reading, which could disadvantage performance on that measure.

The pre- to post-test performance gain of the intervention that combined phonics with shared reading was compared with the mean of the gains of shared reading and explicit phonics interventions, each taught separately. In these comparisons of performance gains, oral reading of isolated words and decoding skill (pseudowords) had substantially greater gains for the combined intervention than the separate interventions. In contrast, the gain in word accuracy in oral text reading was not greater for the combined intervention, failing to reach a statistically significant difference (T&N, Table 5). This orthogonal contrasts analysis, although relevant to the purpose of the study, was not sufficient for this randomized treatments-versus-control design. It also required statistical comparisons between the performance gains of the combined intervention sample and the (math-only) sample that controlled for gains in reading performance from influences external to the intervention. Without these there is no basis to confirm the T&N interpretation that the combined instructional treatment had some significant effects.

Speed of reading was a score in the test of text reading but was not reported, although relevant to comparison of phonics and text-centered instruction (Thompson et al., 2008). And critically, there was no report of the extent to which the children made successful use of the taught explicit phonics in their word responses in text reading, or any of the other reading outcomes. Without this information we are left to speculate whether T&N's claimed (but unconfirmed) positive intervention effects for isolated words and pseudowords could have been an outcome of the children acquiring implicit sublexical processes (Thompson and Fletcher-Flinn, 2012; Thompson, 2014) from the isolated word exemplars for the taught phonics rather than the children's use of the phonics.

Apart from omission of the required statistical comparisons, the design and its implementation in this study may rate above average on a list of validity criteria such as Troia (1999) but our focus has been mainly on ambiguities in validity not often recognized in research on instructional interventions. Included in these are lack of information and evidence for (i) the context of both prior and concurrent instruction, (ii) how the intervention fits wider teaching goals and other instructional needs of the participants, (iii) the extent, and balance, of opportunities in the outcome measures to use procedures that were taught, and (iv) children's use of those procedures in such opportunities, (v) testing contrary predictions from alternative theories.

## Conflict of interest statement

The author declares that the research was conducted in the absence of any commercial or financial relationships that could be construed as a potential conflict of interest.
